# Older age does not influence CD4 cell recovery in HIV-1 infected patients receiving Highly Active Anti Retroviral Therapy

**DOI:** 10.1186/1471-2334-4-46

**Published:** 2004-11-06

**Authors:** Mario Tumbarello, Ricardo Rabagliati, Katleen de Gaetano Donati, Silvia Bertagnolio, Eva Montuori, Enrica Tamburrini, Evelina Tacconelli, Roberto Cauda

**Affiliations:** 1 Department of Infectious Diseases, Catholic University, Rome, Italy; 2 On leave of absence from the Department of Internal Medicine, Pontificia Universidad Catolica de Chile, Santiago, Chile

## Abstract

**Background:**

Diagnosis of HIV infection is recently occurring with increasing frequency in middle-aged and in older individuals. As HAART became available, a minimal beneficial effect on immunological outcome in older in respect of younger subjects has been reported. In fact, both the intensity and the rapidity of the immunological response appeared to be reduced in elderly subjects. On the contrary, only few reports have indicated a similar immunological outcome both in older and younger HIV-positive subjects. Interestingly, older age did not seem to significantly affect the long-term virological outcome of HAART treated subjects.

**Methods:**

To characterise epidemiological and clinical features of older HIV+ subjects, a prospective case-control study was performed: 120 subjects ≥ 50 and 476 between 20 and 35 years were initially compared. Subsequently, to better define the impact of HAART on their viro-immunological response, 81 older were compared with 162 younger subjects.

**Results:**

At baseline cases presented significantly lower TCD4+ cell number and were more frequently affected by comorbid conditions. Under HAART a statistically significant increase in TCD4+ cell number was observed in cases and controls. At multivariate analysis, there was no statistically significant difference between cases and controls regarding viro-immunological response.

**Conclusions:**

Although older subjects present a more severe HIV infection, they can achieve, under HAART, the same viro-immunological success as the younger individuals.

## Background

Early in the epidemic, Human Immunodeficiency Virus (HIV) infection primarily affected young adults; later on, it occurred with increasing frequency in middle-aged and in older individuals, too. In fact, a rate of 12.3% of AIDS patients aged 50 years or older, with subjects ≥ 60 years equalling 3.2%, has been recently reported by the Centres for Diseases Control and Prevention (CDC) [1].

Most of the known epidemiological features about older HIV-infected subjects, have been defined before the introduction of Highly Active Antiretroviral Therapy (HAART), as the standard therapy for HIV-positive subjects, in 1996. A low level of T CD4+ cells at HIV diagnosis [2,3], a rapid decline of T CD4+ cell count [4,5] and a high level of HIV viral load after seroconversion [6,7] have been reported in older HIV-infected subjects. In addition, these individuals presented a clinical condition of AIDS at the time of diagnosis of HIV infection more frequently than younger [2] and they were more likely to die within one month after HIV diagnosis [8]. Several studies have indicated that older patients had a shorter survival than younger [2,9,10], independently of HIV-risk behaviour [5,11,12] and AIDS-related illnesses [13]. Either the more rapid course [4,9] or the decreased survival [10,14] observed in older subjects were considered to be determined by the more frequent presence of non HIV-related comorbidities.

As HAART became available, a less beneficial effect on immunological outcome in older in respect of younger subjects has been reported [15-17]. In fact, both the intensity [15,16] and the rapidity [17] of the immunological response appeared to be reduced in older subjects. On the contrary, only few reports have indicated a similar immunological outcome both in older and younger HIV-positive subjects [18,19]. Interestingly, older age did not seem to significantly affect the long-term virological outcome of HAART treated subjects [15,18,20].

Two were the aims of this study:

1. To characterise the epidemiological and clinical features of older subjects at the time of HIV diagnosis in the HAART era.

2. To better define the impact of HAART on virological and immunological response in a cohort of older HIV-positive patients when confounding variables such as adherence to therapy, side effects and non HIV-related comorbidities were evaluated.

## Methods

### Study setting

The Catholic University teaching hospital is a 1,900-bed tertiary care centre located in Rome, Italy, with approximately 67,000 patient admissions each year. There is a 60-bed unit for the admission of HIV-infected subjects and a day-hospital for out-patient care.

### Study design

Prospective case-control study.

### Study population

All patients presenting the first HIV positive test from January 1997 to June 2003, admitted to our in- and out-patient care, were considered.

For the first aim of the study, subjects aged ≥ 50 years (older) and between 20 and 35 years (younger) were identified.

For the second aim of the study, older and younger patients who were given HAART regularly and with a follow-up of at least six months, were included as cases and controls, respectively (ratio 1:2). The control group was matched by sex, year of HIV diagnosis, presence of AIDS defining conditions and type of HAART regimen (i.e. NNRTI containing vs. PI containing regimens). A patient (either case or control) was considered as regularly HAART-treated if he/she was under antiretroviral therapy with a combination of two nucleoside reverse transcriptase inhibitors (NRTI) plus either a non nucleoside reverse transcriptase inhibitor (NNRTI) or a protease inhibitor (PI), as single drug or with the addiction of a low dose of ritonavir (i.e. 100 mg bid), for at least three months.

### Parameters evaluated

Patients' records were reviewed by one of us, using a standardised data collection form with predefined criteria. For each subject enrolled in the study, the following data were obtained at inclusion after patient's informed consent: age, gender, HIV-risk behaviour, date of the first available HIV test, reason for HIV testing, CDC stage of HIV infection [21], HIV-related conditions, date of AIDS diagnosis, number of hospitalisations after HIV diagnosis, observed AIDS free interval (i.e. time from HIV infection to AIDS diagnosis), type and use of prophylaxis for *Pneumocystis carinii *pneumonia (PCP) and toxoplasmosis, antiretroviral therapy (type, duration, adherence and side effects) and other medications (antituberculous drugs, antivirals, corticosteroids, antacids), time of follow-up, time from AIDS diagnosis to death, cause of death.

Data collected from the laboratory records included: numbers of circulating CD4+ T cells (/mm^3^), CD8+ T cells (/mm^3^) and plasma HIV RNA viral load (copies/ml) with a limit of detection below 50 copies/ml. The time of the beginning of HAART was considered as time zero in the analysis. All subjects underwent periodical clinical evaluations including determination of CD4+ T cells and HIV viral load every six months.

Adherence was defined through a self-reported evaluation by the patient and registered as percentage by the physician. An evaluation higher than 80% was classified as "adherent", whereas lower than 80% was considered as "non-adherent". The following adverse effects were considered: anaemia (haemoglobin <9.0 gr/dl), digestive intolerance, dyslipidemia (serum cholesterol >240 mg/dl and/or triglycerides >200 mg/dl), hepatitis, hyperglycaemia (glucose >150 mg/dl), lipodystrophy, pancreatitis, peripheral neuropathy, rash, renal colic and thrombocytopenia (platelets <50,000 cells/mm^3^).

Three outcomes were considered for the study: 1. Immunological success (IS): defined as T lymphocytes CD4+ cell count >200/mm^3 ^at the end of the follow-up; 2. Virological success (VS) defined as a HIV viral load undetectable (<50 copies/ml) at the end of the follow-up; 3. Viro-immunological success (VIS) defined as either undetectable HIV viral load and T lymphocytes CD4+ cell count >200/mm^3 ^at the end of the follow-up.

### Evaluation of comorbidity

In order to evaluate the significance of non HIV-related comorbid conditions, we used a modified version of the Charlson comorbidity index which excludes AIDS from the diagnosis [22]. Cardiovascular, pulmonary, neurological, endocrine, renal, hepatic, gastrointestinal, rheumatologic diseases, coagulation disorders and cancer were considered as comorbid non HIV-related conditions.

### Statistical analysis

Quantitative variables were tested for normal distribution and compared by means of Student's two tailed t-test. Differences in group proportions were assessed by use of the χ^2 ^test or, for small numbers, Fisher's exact test. Stepwise logistic regression models were used for each factor to adjust for the effects of confounding variables. Two tailed tests of significance at the p < 0.05 level were used to determine statistical significance. Statistical analysis was performed using the software program Intercooled Stata 6.0 (Stata Corporation, Texas).

## Results

### Epidemiological and clinical features of older patients at the time of HIV diagnosis

The rate of HIV diagnosis in patients aged ≥ 50 years admitted to our in- and out-patient care remained constant (about 10%) over the study-period.

From a total of 1265 subjects with new HIV-positive test, 129 (10,1%) subjects aged ≥ 50 years were identified. Nine of them were not further considered because of missing data. One hundred twenty older subjects were evaluated. Data were available for 476 younger subjects.

Among older subjects there were more males, less intravenous drug abusers, more advanced HIV disease, more frequent C stage of HIV infection and higher mortality, (Table 1).

The reasons for the first HIV test were comparable both for older and younger subjects. In particular, 30 (25%) older subjects were asymptomatic. For those who were symptomatic, the more frequently observed symptoms were: fever (25%), haematological dysfunctions (17%), weight loss (14%), neurological (8%), gastrointestinal (6%) or hepatic (7%) disorders.

Regarding AIDS-defining conditions, a significantly higher frequency of PCP, HIV-encephalopathy, Kaposi's sarcoma and cryptococcosis was observed in older HIV-infected patients than in younger.

### Matched case-control study

Thirty nine out of 120 HIV-positive older subjects were not further considered: 29 because the duration of follow-up was below six months and ten because the case-control matching was not possible. A final number of 81 older patients under regular HAART and with a follow-up of at least six months (cases) were thus compared with 162 younger subjects (controls).

#### Baseline characteristics and evolution

Cases had a median age of 56.9 ± 5.2 years and controls of 31.3 ± 3.2 years. As a result of the matching, 73% of subjects in both groups were males and 49% were in CDC stage C of HIV infection. HIV infection risk factors were similar in the two groups, but eighteen (22%) cases referred an unknown risk factor compared to 26 (16%) controls (p = 0.1). The mean of CD4+ T cells was significantly lower in cases compared to controls (107 ± 109 cell/mm^3 ^vs. 178 ± 189 cell/mm^3^; p < 0.01), such as the mean CD4 percentage (9% vs. 14%; p < 0.01). The mean of CD8+ T cells at baseline was similar in cases and controls (757 ± 380 cell/mm^3 ^vs. 818 ± 362) (p = ns). Mean of HIV viral load log was similar in the two groups (4.65 ± 0.57 vs. 4.74 ± 0.76 copies/ml; p = ns). No statistical significant difference was observed for haematological parameters, serological markers of previous viral hepatitis (type B or C) and syphilis. Laboratory tests only indicated a significant increase of glycaemia in older patients (103.3 ± 18.7 vs. 89.0 ± 10.6 mg/dl; p < 0.01).

Follow-up was similar in the two groups (40 ± 13 vs. 42 ± 11 months; p = ns), ranging from 6 to 78 months.

No differences were observed between cases and controls in the number of hospitalisations (1.2 ± 2.1 vs. 1.3 ± 2.8; p = ns).

Seven (9%) and six (4%) patients died in the case and control groups, respectively (p = ns). All causes of death in both groups were HIV-related conditions with the exception of one case who died for an acute mesenteric vascular episode.

#### Comorbid conditions

Cases had more comorbid conditions than controls (45.6% vs. 15.9%; p < 0.01). One third of the cases suffered from cardiovascular diseases, a rare condition among controls (32.2% vs. 2.1%; p < 0.01). Gastrointestinal disorders (9.4% vs. 1.2%; p = 0.04) and diabetes (11.0% vs. 2.1%; p = 0.02) were also significantly more frequent among cases than controls. No statistically significant differences were found for cancer, pulmonary, renal or endocrine diseases, chronic hepatitis and coagulation disorders. The total comorbid points were 37 for cases and 20 for controls. Cases had also a higher mean Charlson index in respect to controls (0.57 ± 0.92 vs. 0.12 ± 0.44; p < 0.01).

#### Antiretroviral therapy

No statistically significant difference between cases and controls was observed in type, number and duration of HAART regimens. The first line antiretroviral therapy included PI in more than 80% of cases and controls (Table 2). The patients who required a change in antiretroviral therapy (second line therapy) received a therapeutical cocktail including PI in 40% vs. 48% (p = ns) and NNRTI in 57% vs. 50% (p = ns) of cases and controls, respectively. Similar percentages were observed in the patients who required another change of antiretroviral therapy (third line therapy) during the follow-up. In the first line drug regimen, side effects were more frequently responsible of the change of the drug regimen in cases compared with controls, although not reaching a statistically significant level (p = 0.1). A high level of adherence to HAART was observed in both groups (82% vs. 75%; p = ns). The more frequent adverse reactions were dyslipidemia, digestive intolerance and lipodystrophy in both groups (p = ns). A significantly higher frequency of hyperglycaemia and anaemia was observed in cases than in controls (Table 2).

#### Immunological and virological response

##### HAART related immunoreconstitution

**Figure 1:**
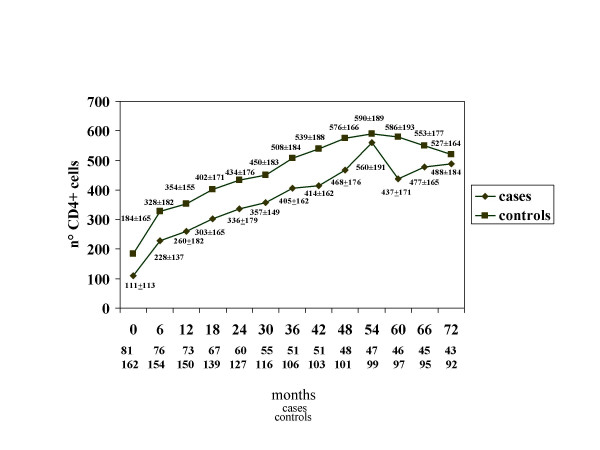
T CD4+ cell count/mm^3 ^(mean ± SD) at the beginning of HAART and each six months of follow-up in cases and controls.

Comparing the mean values obtained at baseline in both cases and controls with those obtained at the end of the follow-up, a statistically significant increase in T CD4+ cell number was observed. In particular, T CD4+ cell count increased in 48 months from 107 ± 109 to 476 ± 258 for cases (p = 0.04), and from 178 ± 189 to 584 ± 252 for controls (p < 0.01). There was always a progressive linear enhancement in both groups at any individual time of the follow-up. The lower values observed in cases in respect to controls at baseline, persisted until the end of the follow-up (Figure 1).

**Figure 2:**
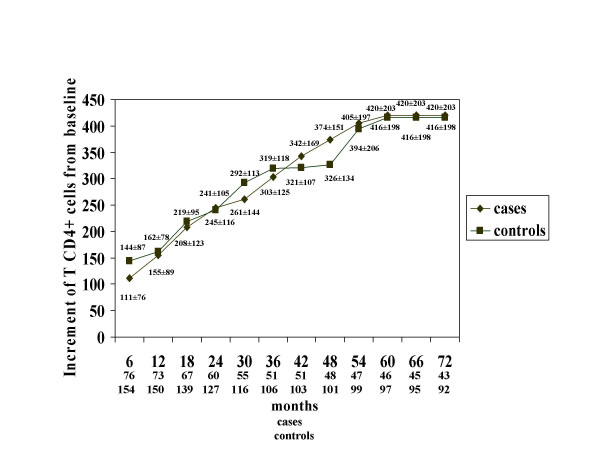
T CD4+ cell count/mmc six month increment (mean ± SD) from the beginning of HAART to the end of the follow-up in cases and controls

The increasing value of T CD4+ cell count every six months in respect to baseline was comparable in the two groups (Figure 2).

According to the above mentioned outcome definition, IS was observed in 59 (73%) of the cases and 128 (79%) of the controls (p = ns). The following variables were significantly associated with IS at univariate analysis: CDC stage A (p = 0.01), low Charlson index (p = 0.01), CD4+ T cell count at the beginning and at 6,12,18,24,30,36 months of HAART (p < 0.01), months to achieve CD4+ T cell >200/mm^3 ^(p < 0.01), enhancement of CD4+ T cell count in the first six months (p < 0.01), HIV viremia at 30 months (p < 0.01).

##### HAART related viral suppression

A statistically significant reduction of HIV viral load was observed in both cases and controls when comparing baseline with the end of the follow-up values. In particular, means of HIV-RNA viral load log decreased in 48 months from 4.74 ± 0.57 to 2.01 ± 0.4 copies/ml in cases (p < 0.01) and from 4.69 ± 0.76 to 1.6 ± 0.2 copies/ml in controls (p < 0.01).

**Figure 3:**
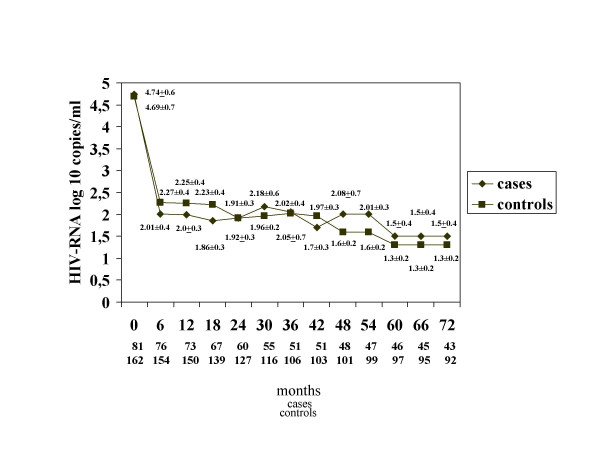
Comparison of HIV-RNA log 10 (mean ± SD) between cases and controls from baseline to the end of follow-up.

HIV viral load reduced under antiretroviral therapy in both groups in a comparable manner. In fact, there was no statistically significant difference between cases and controls at any individual time of the follow-up (Figure 3).

According to the above mentioned outcome definition, VS was observed in 66 (82%) of cases and 121 (75%) of controls (p = ns). The following variables were significantly associated with VS at univariate analysis: CDC stage A (p = 0.02), high adherence to HAART (p = 0.01), dyslipidemia as adverse effect of HAART (p = 0.01), HIV viral load at 6 (p < 0.01) and 24 months (p = 0.02).

##### HAART related virological and immunological outcome

According to our definition of the outcome, VIS was observed in 53 (66%) of cases and 100 (62%) of controls (p = ns). The following variables were significantly associated with VIS at univariate analysis: CDC stage A (p < 0.01), high adherence to HAART (p = 0.02), dyslipidemia as adverse effect of HAART (p < 0.01), CD4+ T cell number at baseline and at 6,12,18,24,30,36 months of HAART (p < 0.01), enhancement of CD4+ T cellcount in the first six months (p < 0.01), HIV viral load at 6 (p = 0.04) and 30 months (p = 0.01).

No statistical difference in immuno-virological response was found between cases and controls when, as cases, only a subgroup of patients aged > 60 years was considered (data not shown).

#### Multivariate analysis

In a multivariate logistic regression model, after adjustment for sex and Charlson index, there was no statistically significant difference between cases and controls for IS, VS and VIS (p = ns). Similar results were obtained when CDC stage A and CD4+ T cell count at HIV diagnosis were added to the model.

Another model including sex, Charlson index, CDC stage A, CD4+ T cell count at the beginning of HAART, adherence and dyslipidemia as adverse effect of HAART did not show any statistically significant difference between cases and controls for IS, VS and VIS.

Further adjustment for sex, Charlson index, CDC stage A, adherence, dyslipidemia as adverse effect of HAART, CD4+ T cell count at beginning of HAART and after six months of therapy, enhancement of CD4+ T cell count in the first six months, months to achieve CD4 + T cell count >200/mm^3^, HIV viral load at six months did not modify the results.

## Discussion

This study, conducted in an Italian population, shows that in the HAART era, the percentage of older patients out of the new HIV infections is approximately 10%, slightly higher than previously reported [23]. The impact of long term HAART-related immunoreconstitution in this sub-population of HIV patients could reduce the incidence of older patients with AIDS within the next years to less than the 12% recently reported [1].

The first part of our study confirms previous pre-HAART era observations, about more severe HIV infection in older subjects [2-10]. In fact, higher frequency of patients with AIDS at HIV diagnosis, lower T CD4+ cell count and higher mortality in older subjects in respect of younger were observed. A late diagnosis or a more aggressive impact of HIV infection in older population could explain these results. The first possibility could reflect low level information and/or physicians' insufficient suspicion [2,24], despite the world-wide durable informative campaigns and preventive efforts on this issue. Alternatively, a shorter or less symptomatic pre-AIDS phase, as already reported in older HIV-positive subjects, could contribute to the late diagnosis [2]. On the other hand, the more aggressive impact of HIV infection in older population has been related to the hypothesis that HIV is an additional aggravating factor to an already debilitated host potentially presenting numerous comorbidities, less physiological reserves [10,14] and a less responsive immune system [2,25]. In fact, in HIV-negative older subjects, important changes in cellular and humoral components of the immune system including defects in T CD4+ lymphocyte function, have been described [26].

Regarding HIV-risk behaviour, our data indicate the heterosexual route as the most frequent in older patients, followed by no risk reported, homosexual contacts, intravenous drug abuse. In particular, our data confirm recent reports [8,27] indicating an increased incidence of HIV subjects with no risk reported. This aspect could contribute to the above mentioned low level of suspicion of HIV diagnosis in older subjects. It is of interest that a large epidemiological survey in the US general population has reported a prevalence of at least one risk factor for HIV infection in about 10% of subjects aged 50 years or older [28].

To date, no studies have indicated that older AIDS patients should be treated differently for HIV disease. Clinical drug trials often exclude older patients because of multiple medical problems, comorbidities or co-existing non HIV-related medication regimens [29]. The first available literature data obtained in a large population of HIV-positive patients treated with zidovudine in the early nineties showed that age was an independent predictor of rapid progression to AIDS and shorter survival [30]. On the contrary, antiretroviral therapy was the only independent predictor of survival after the diagnosis of AIDS in older HIV-positive subjects in the pre HAART era [31].

Very little literature and study has been devoted specifically to the role of protease inhibitors and aggressive antiretroviral triple therapy in elderly patients. Few data have been published concerning the specific response of older patients to new HIV antiretroviral treatments, and currently there are no guidelines for specific antiretroviral treatment modalities for patients>50 years of age. However, a poor immunological response under HAART was observed in small groups of patients [15,16] possibly due to the well known depressed thymic function of the elderlies [15-17]. Only few reports showed a similar virological and immunological outcome in older HIV-positive compared with younger subjects [18,19].

This six year prospective matched case control study not only confirms this observation but indicates that older patients under HAART experienced a successful immunological response comparable to younger subjects also in a long term follow-up, in line with preliminary reports [32-34]. In fact, our older patients, starting HAART with a lower level of T CD4+ cells than younger, obtained a significant increment in their T CD4+ cell count, with the same rapidity, intensity and persistence in time as observed in younger patients.

Meticulous adherence to HAART is necessary to achieve an optimal clinical and virological response. A variety of factors may predict medication adherence among HIV-infected adults. Although older age is usually associated with higher rates of antiretroviral adherence, older participants who were cognitively impaired could have difficulty in adequately adhering to their medication regimen [35-37]. Our findings indicate that this goal could be achieved independently from age. In fact, although thymic functions decline with age, substantial output of T CD4+ cells can be maintained into late adulthood, thus contributing to HAART-related immune recovery in older HIV-positive patients [38-40].

Early work also demonstrated a positive association between age and the plasma HIV-1-RNA copy number at the time of identification of HIV-1 serostatus among recently diagnosed older HIV-1 positive individuals [41]. However, this could have been caused by a lead-time diagnostic bias, with a greater delay among older individuals.

No differences were observed in virological response as indicated by viral load reduction in older patients with respect to younger. This result is in line with previous reports [15,18] including a large study which indicated that older age decreases the risk to have viral load greater than 1000 copies/ml in HIV-positive subjects under HAART [2]. In a recent report, older age was associated with lower levels of HIV-1 replication, independent of antiretroviral therapy usage, regimen adherence, and diseases stage. It is suggested that the effect may be caused by changes in viral evolution or immunological monitoring specific to older individuals with HIV-1 infection [42].

In our study both the frequency and the distribution of adverse effects of HAART were similar between the two groups, with the exception of anaemia and hyperglycaemia which were more frequent in older patients. Hyperglycaemia can be explained because diabetes is a frequent comorbid condition among older. Older subjects were more susceptible than younger to adverse effects in the first line therapy; consequently they had to change therapy more frequently.

Univariate analysis showed an association of low Charlson index with immunological success. However, considering that the Charlson index was higher in older patients, none of the models of multivariate analysis indicated an association between comorbidity and virological and/or immunological response. Nonetheless previous studies have suggested a more rapid course and a decreased survival in older HIV-positive patients because of the presence of comorbidities [10], our data did not confirm this association (data not shown). Interestingly, cardiovascular disease and diabetes were the more frequent comorbidities in our older population.

Dyslipidemia and lipodystrophy in association with gastrointestinal disorders were the more frequent adverse reactions. In view of these results, of the recent reports of endothelial vascular damage, premature atherosclerosis and coronary events in younger HAART-treated patients [43-45], an important increase of cardiovascular event incidence in older HIV-positive patients under HAART could not be excluded to occur in the forthcoming years.

## Conclusions

In conclusion, we strongly underline the need for an early diagnosis of HIV infection in older subjects through implementation of educational campaigns specifically targeted to this population. In relation to older people, physicians should consider more often the possibility of HIV diagnosis, through more screening and counselling. An early diagnosis is mandatory because, although older subjects present with more severe HIV infection, the use of HAART allow them to achieve the same viro-immunological response as younger individuals. At present, there is an increasing number of older subjects living with HIV either because of new HIV diagnosis in older population or because who have previously acquired HIV infection now become older due to HAART improved survival. It is reasonable to predict that the average age of chronically treated HIV-positive patients will progressively increase in the forthcoming years. Based on this reality, ageing with HIV is a newly manifested chronic disease with a complex long-term management in consideration also of the impact of HIV and HAART on the natural history of other chronic diseases typically associated with older age.

## List of abbreviations

CDC Centers for Diseases Control

HAART Highly Active Antiretroviral Therapy

NRTI Nucleoside Reverse Trascriptase Inhibitor

NNRTI Non Nucleoside Reverse Trascriptase Inhibitor

PI Protease Inhibitor

VIS Viro Immunological Success

VS Virological Success

IS Immunological Success

## Competing interests

The author(s) declare that they have no competing interests.

## Authors' contributions

**MT **conceived the study, performed the statistical analysis and wrote the manuscript

**RR **participated in the design of the study, in the collection of the data, in the statistical analysis and in the writing of the manuscript

**KdGD **participated in the design of the study, in the collection of the data and in the writing of the manuscript

**SB **participated in the design of the study and in the collection of the data

**EM **participated in the collection of the data

**ET **participated in the collection of the data

**ET **participated in the design of the study

**RC **did the supervision and critically read the last version of the paper

All authors read and approved the final manuscript

## Pre-publication history

The pre-publication history for this paper can be accessed here:



## Figures and Tables

**Table 1:** Demographic and clinical characteristics of older (≥ 50 years) and younger (20–35 years) HIV-positive subjects in the period 1997–2003.

	Older n = 120 (%)	Younger n = 476 (%)	p
Male sex	91(76)	285 (60)	<0.01
Age (mean ± SD), years	58 ± 5.1	31 ± 3.2	<0.01
Risk factor of HIV infection			
Heterosexual	62(52)	199(42)	0.1
No risk reported	32(27)	119 (25)	0.6
Homosexual	19(16)	57 (12)	0.2
Injection drug users	6(5)	104(22)	<0.01
Months between first HIV+ test and AIDS (mean ± SD)	1.8 ± 7.8	3.3 ± 6.7	0.4
Stage C of HIV infection*	62 (52)	114 (24)	<0.01
PCP	9 (8)	14 (3)	0.02
HIV encephalopathy	9 (8)	9 (2)	<0.01
Kaposi	8 (7)	5 (1)	<0.01
Candidiasis	8 (7)	43 (9)	0.5
Cryptococcosis	5 (4)	1 (0.3)	<0.01
Mycobacteriosis	4 (3)	5 (1)	0.2
Cryptosporidiosis	4 (3)	5 (1)	0.06
Tuberculosis	4 (3)	14 (3)	0.7
Isosporiasis	1 (1)	0	0.2
Bacterial pneumonia	1 (1)	0	0.2
Wasting syndrome	1 (1)	9 (2)	0.5
Toxoplasmosis	0	9 (2)	0.1
Cytomegalovirus retinitis	0	1 (0.3)	0.6
Lymphoma	7 (6)	9 (2)	0.05
CD4+ T cell/mm^3 ^(mean ± SD)**	59.1 ± 65.3	105.3 ± 127.4	0.04
Deaths	16 (13)	14 (3)	<0.01

*see Ref. n° 21; **at AIDS diagnosis;

**Table 2:** Antiretroviral therapy in 81 cases and 162 controls

	Cases n (%)	Controls n (%)	p
First line therapy	81 (100)	162 (100)	
Combinations			
-NRTI+PI	67 (83)	138 (85)	0.7
-NRTI+NNRTI	14 (17)	26 (16)	0.7
Duration; months (mean ± SD)	13.46 ± 10.23	16.67 ± 8.45	0.06
N° of patients who suspended	53 (66)	91 (56)	0.2
Cause of suspension			
-side effects	30 (56)	37 (41)	0.1
-viro-immunological failure	8 (15)	15 (17)	0.6
Second line therapy	45 (56)	91 (56)	1
Third line therapy	11 (14)	31 (19)	0.4
Total number of drug changes (mean ± SD)	2.1 ± 0.9	2.0 ± 0.6	0.8,
Adherent to the last drug regimen	68 (84)	118 (73)	0.08
Side effects to HAART			
dyslipidemia	32 (40)	55 (34)	0.5
digestive intolerance	26 (32)	58 (36)	0.6
lipodystrophy	12 (15)	23 (14)	0.7
hyperglycaemia	8 (10)	3 (2)	0.01
anaemia	8 (10)	3 (2)	0.01
